# Inflammatory monocyte gene expression: trait or state marker in bipolar disorder?

**DOI:** 10.1186/s40345-015-0037-x

**Published:** 2015-09-17

**Authors:** K. Becking, B. C. M. Haarman, R. F. Riemersma van der Lek, L. Grosse, W. A. Nolen, S. Claes, H. A. Drexhage, R. A. Schoevers

**Affiliations:** ICPE/UCP/Triade (CC.72), Interdisciplinary Center Psychopathology and Emotion Regulation (ICPE), University Medical Center Groningen, University of Groningen, PO Box 30001, 9700 RB Groningen, The Netherlands; Department of Psychiatry, University Medical Center Groningen, University of Groningen, Groningen, The Netherlands; Radiology Morphological Solutions, Berkel en Rodenrijs, The Netherlands; Department of Psychiatry, University of Münster, Münster, Germany; Department of Psychiatry, University of Leuven (KU Leuven), Louvain, Belgium; Department of Immunology, Erasmus MC, Rotterdam, The Netherlands

**Keywords:** Bipolar disorder, Gene expression, Mood episode, Trait, State

## Abstract

**Background:**

This study aimed to examine whether inflammatory gene expression was a trait or a state marker in patients with bipolar disorder (BD).

**Methods:**

69 healthy controls (HC), 82 euthymic BD patients and 8 BD patients with a mood episode (7 depressed, 1 manic) were included from the MOODINFLAME study. Six of the eight patients who had a mood episode were also investigated when they were euthymic (6 of the 82 euthymic patients). Of these participants the expression of 35 inflammatory genes was determined in monocytes using quantitative-polymerase chain reaction, of which a total gene expression score was calculated as well as a gene expression score per sub-cluster.

**Results:**

There were no significant differences in inflammatory monocyte gene expression between healthy controls and euthymic patients. Patients experiencing a mood episode, however, had a significantly higher total gene expression score (10.63 ± 2.58) compared to healthy controls (*p* = .004) and euthymic patients (*p* = .009), as well as when compared to their own scores when they were euthymic (*p* = .02). This applied in particular for the sub-cluster 1 gene expression score, but not for the sub-cluster 2 gene expression score.

**Conclusions:**

Our study indicates that in BD inflammatory monocyte, gene expression is especially elevated while in a mood episode compared to being euthymic.

## Background

Disturbances in the immune system have frequently been reported in bipolar disorder (BD) (Leboyer et al. 2012). Several meta-analyses found peripheral cytokines to be raised in patients compared to healthy controls (HC) (Modabbernia et al. 2013; Munkholm et al. 2013). However, the results are heterogeneous, with also studies reporting on normal (Guloksuz et al. 2010) or even lower cytokine levels (Boufidou et al. 2004) in BD compared to HC. This may be due to the fact that peripheral cytokines are strongly influenced by lifestyle and disease factors (O’Connor et al. 2009). Focusing on the main cellular producers of these cytokines, such as circulating monocytes and macrophages, may be a better approach to find stable markers for BD. Indeed, studies from our group focusing on gene expression of circulating monocytes found a discriminating pro-inflammatory gene expression in BD patients compared to HC (Drexhage et al. 2010; Padmos et al. 2008).

It remains unclear whether these immunological disturbances are related to the mood state, or are a trait phenomenon. Most studies compared BD patients to HC, without differentiating between patients in different mood states. The few available studies that examined immune disturbances across mood states found significantly higher levels of peripheral inflammatory markers during a mood episode compared to euthymia (Barbosa et al. 2014; Brietzke et al. 2009; Cunha et al. 2008; Ortiz-Domínguez et al. 2007; Tsai et al. 2012). Regarding inflammatory gene expression, our original hypothesis prior to the study described below was that monocyte activity might be a diagnostic biomarker for BD and thus a trait factor. However, in further analysis of our previous study, we already found the expression of specific inflammatory genes to be higher in a small subsample of depressed versus euthymic patients and to a lesser extent in manic compared to euthymic patients (Padmos et al. 2008). Furthermore, we reported a possible relation between a sub-cluster of genes and manic symptomatology (B.C.M. Haarman et al. 2014a) in BD.

In this report, we present the results of the MOODINFLAME study, in which we compared euthymic BD patients with HC. Moreover, we present the results in of a small additional study in which BD patients were compared both in a mood episode and when euthymic. Thus, our study aimed to examine whether inflammatory gene expression in monocytes is a trait or a state marker in BD.

## Methods

### Participants

Data were derived from the EU-funded MOODINFLAME study (“MOODINFLAME website” 2014) carried out to investigate possible inflammatory biomarkers to advance early diagnosis, treatment and prevention of mood disorders. In the MOODINFLAME study, adult male and female subjects were included who were free of inflammation-related symptoms including fever and current or recent infectious or inflammatory disease, uncontrolled systemic disease, uncontrolled metabolic disease or other significant uncontrolled somatic disorders known to affect mood. They did not use somatic medication known to affect mood or the immune system, such as corticosteroids, non-steroid anti-inflammatory drugs and statins. Female candidates who were pregnant or recently gave birth were excluded. The present study has been set up as a cross-sectional case–control study extended with a within-patient longitudinal design. Blood was analyzed of a sample of 159 adult participants recruited from two university psychiatry clinics in Groningen (The Netherlands) and Leuven (Belgium). The sample consisted of 69 HC, 82 euthymic BD patients (BD-Eu) and 8 BD patients with a mood episode (BD-Ep) (seven depressed, one manic). Six of the eight BD-Ep patients were also investigated when they were euthymic (6 of the 82 BD-Eu patients). Of these patients, one patient was first sampled in an episode and resampled after he was recovered, and the remaining five were first sampled being euthymic and resampled when they became ill again.

The study was approved by the ethical committees of the participating universities, and written informed consent was obtained from all participants.

### Assessments

DSM-IV BD diagnoses were established using the Mini-International Neuropsychiatric Interview (MINI) (Lecrubier et al. 1997; Sheenan et al. 1998). The severity of depression was measured by the Inventory of Depressive Symptoms (IDS-C_30_) (Rush et al. 1996) for BD patients in a face-to-face interview, for HCs with a self-report questionnaire (IDS-SR_30_). To determine the presence or intensity of manic symptomatology in patients, the Young Mania Rating Scale (YMRS) (Young et al. 1978) was used. Mood states were defined as euthymic, manic or depressed, based on the MINI. BD-Eu patients were neither in a depressed nor (hypo-)manic episode at the time of measurement as indicated by an IDS-C_30_ score <22 and a YMRS score <12, respectively. The remaining clinical characteristics were obtained with the Patient Questionnaire from the former Stanley Foundation Bipolar Network, including separate clinician and patient chapters covering a spectrum of clinical features (Leverich et al. 2001). In the event of a mismatch of results from the MINI in relation to the Patient Questionnaire, diagnoses were checked with the treating physician. Age of onset was defined as the age when the first mood episode occurred, and information on psychiatric medication was dichotomized.

### Laboratory methods

To detect the expression of inflammatory genes of monocytes, similar methods were used as described in the original study by Padmos et al. (2008). In short, RNA was isolated from purified monocytes and to obtain c-DNA for quantitative-polymerase chain reaction (q-PCR), 1 μg of RNA was reverse transcribed using the cDNA high-capacity cDNA Reverse Transcription Kit (Applied Biosystems, Carlsbad, CA, USA). Then, relative to the housekeeping gene ABL1, the expression of ADM, ATF3, BCL2A1, BTG3, CCL2, CCL20, CCL7, CD9, CDC42, CXCL2, DHRS3, DUSP2, EMP1, EREG, FABP5, HSPA1A/HSPA1B, IL-1α, IL-1β, IL1R1, IL-6, IRAK2, MAFF, MAPK6, MXD1, NAB2, PDE4B, PTGS2, PTPN7, PTX3, RGCC32, SERPINB2, STX1A, THBD, TNF and TNFAIP3 was determined, using the comparative threshold cycle (CT) method (Biosystems 2001). See Table 1 for the list of genes and corresponding proteins. Data were expressed as ΔCT values (values corrected to ABL1) and to control for site (Groningen and Leuven), fold change transformation was applied. By dividing the ΔCT scores of patients from Groningen by the mean of healthy controls from Groningen and subsequently the scores of patients from Leuven by the mean of healthy controls from Leuven, the relative gene expression was expressed as a fold change (FC) value (Biosystems 2001).

**Table 1:** List of genes with corresponding proteins

Gene symbol	Name of corresponding protein
Inflammation	
ATF3	Cyclic AMP-dependent transcription factor 3
BCL2A1	B cell lymphoma-2-related protein A1
CCL20	C–C chemokine ligand 20
CXCL2	C–X–C chemokine ligand 2
DUSP2	Dual specificity protein phosphatase 2
EREG	Epiregulin
IL-1β	Interleukin 1β
IL-6	Interleukin 6
PDE4B	cAMP-specific 3′,5′-cyclic phosphodiesterase 4B
PTGS2	Prostaglandin G/H synthase (cyclooxygenase)
PTX3	Pentraxin-related protein 3
TNF	Tumor necrosis factor
TNFAIP3	Tumor necrosis factor, alpha-induced protein 3
Chemotaxis/adhesion/differentiation/motility	
CCL2	C–C chemokine ligand 2
CCL7	C–C chemokine ligand 7
CDC42	Cell division control protein 42 homolog
DHRS3	Short-chain dehydrogenase/reductase 3
EMP1	Epithelial membrane protein 1
MAPK6	Mitogen-activated protein kinase 6
NAB2	Nerve growth factor-induced protein A-binding protein 2
PTPN7	Protein tyrosine phosphatase, non-receptor type 7
STX1A	Syntaxin-1A
Other	
ADM	Adrenomedullin
BTG3	BTG family, member 3
CD9	Cluster of differentiation 9 antigen
FABP5	Fatty acid-binding protein 5
HSPA1/HSPA1B	Heat shock 70 kDa protein 1
IL-1α	Interleukin 1α
IL1R1	Interleukin 1 receptor, type 1
IRAK2	Interleukin 1 receptor-associated kinase-like 2
MAFF	Musculoaponeurotic fibrosarcoma oncogene homolog F
MXD1	MAD protein
RGCC32	Regulator of cell cycle
SERPINB2	Plasminogen activator inhibitor-2
THBD	Thrombomodulin

### Gene score calculation

To obtain a simple measure for overall monocyte activation, we calculated a gene score from the expression levels as described by Grosse et al. (2014). For each of the 35 genes, we determined a range in HC fold change gene expression, defined by the HC mean ± 1 standard deviation (SD). Then, we used this range as a standard to compare the gene expression across the different groups. A gene was considered up-regulated if the FC value was higher than the HC mean + 1 SD, and down-regulated if the FC-value was lower than the HC mean − 1 SD. Then, we calculated a total gene expression score by adding all up-regulated (+1), all down-regulated (−1) and all normally expressed (0) genes for each patient. This method proved to be valid, since the total gene scores showed highly significant correlations with the majority of the genes (Grosse et al. 2014).

Additionally, we calculated two separate sub-cluster gene scores, based on previous cluster analyses performed by Drexhage et al. (2010). The first sub-cluster consisted primarily of pro-inflammatory genes (see Table 1) and the second sub-cluster consisted of chemotaxis, adhesion, differentiation and motility genes (see Table 1).

### Statistical analyses

All data were analyzed with SPSS version 20.0 (SPSS, Chicago, IL, USA). Sample characteristics were compared using Pearson’s Chi square and Fisher’s exact tests for dichotomous and categorical variables, and for continuous variables ANOVA and *t* tests were used. To compare inflammatory gene expression scores across HCs, euthymic patients, mood episode patients and within-patient analyses, ANOVA was used. Results were reported as mean ± standard error. Because only an overall inflammatory gene expression score was used, correction for multiple testing was not applied. As a set of sensitivity analyses, we repeated all analyses using ANCOVA controlling for sex, age and body mass index (BMI).

**Table 2:** Characteristics of patients and healthy controls (*N* = 159)

	Healthy controls (*N* = 69)	Bipolar disorder (*N* = 90)		*p* ^a^
		Euthymic (*N* = 82)	Mood episode (*N* = 8)	
Female, n (%)	39 (56.5)	41 (50.0)	5 (62.5)	.63
Age, mean (SD)	44.7 (16.1)	43.1 (12.1)	41.8 (12.7)	.72
BMI, mean (SD)	23.9 (3.2)	25.7 (4.2)	26.89 (4.2)	*.005*
IDS score, mean (SD)	4.8 (3.4)	8.7 (8.0)	42.1 (14.1)	*<.001*
Clinical characteristics				
YMRS score, mean (SD)	–	1.3 (1.2)	4.7 (6.4)	*.001*
Bipolar I disorder, *n* (%)	–	53 (64.4)	5 (62.5)	.90
Bipolar II disorder, *n* (%)	–	29 (35.4)	3 (37.5)	
Age of onset, mean (SD)	–	23.3 (9.6)	21.8 (9.1)	.68
Lifetime psychotic features, *n* (%)		26 (31.7)	2 (25.0)	.70
Psychotropic medication	–			
Melatonin	–	1 (1.2)	2 (25.0)	*.02*
SSRI	–	4 (4.9)	1 (12.5)	.38
Antipsychotics	–	14 (17.1)	1 (12.5)	.74
Lithium	–	62 (75.6)	2 (25.0)	*.003*
Benzodiazepines	–	11 (13.4)	2 (25.0)	.37
Antiepileptics	–	22 (26.8)	2 (25.0)	.91

Differences (italics values) were considered significant if *p* < .05

*SD* standard deviation, *BMI* body mass index, *IDS* Inventory of Depressive Symptoms, *YMRS* Young Mania Rating Scale, *SSRI* selective serotonin reuptake inhibitor

^a^Based on *χ* ^2^ tests and Fisher’s exact tests for dichotomous and categorical variables, ANOVA tests when comparing age and BMI, and *t* tests when comparing continuous variables between euthymic and mood episode BD patients

## Results

**Fig. 1:**
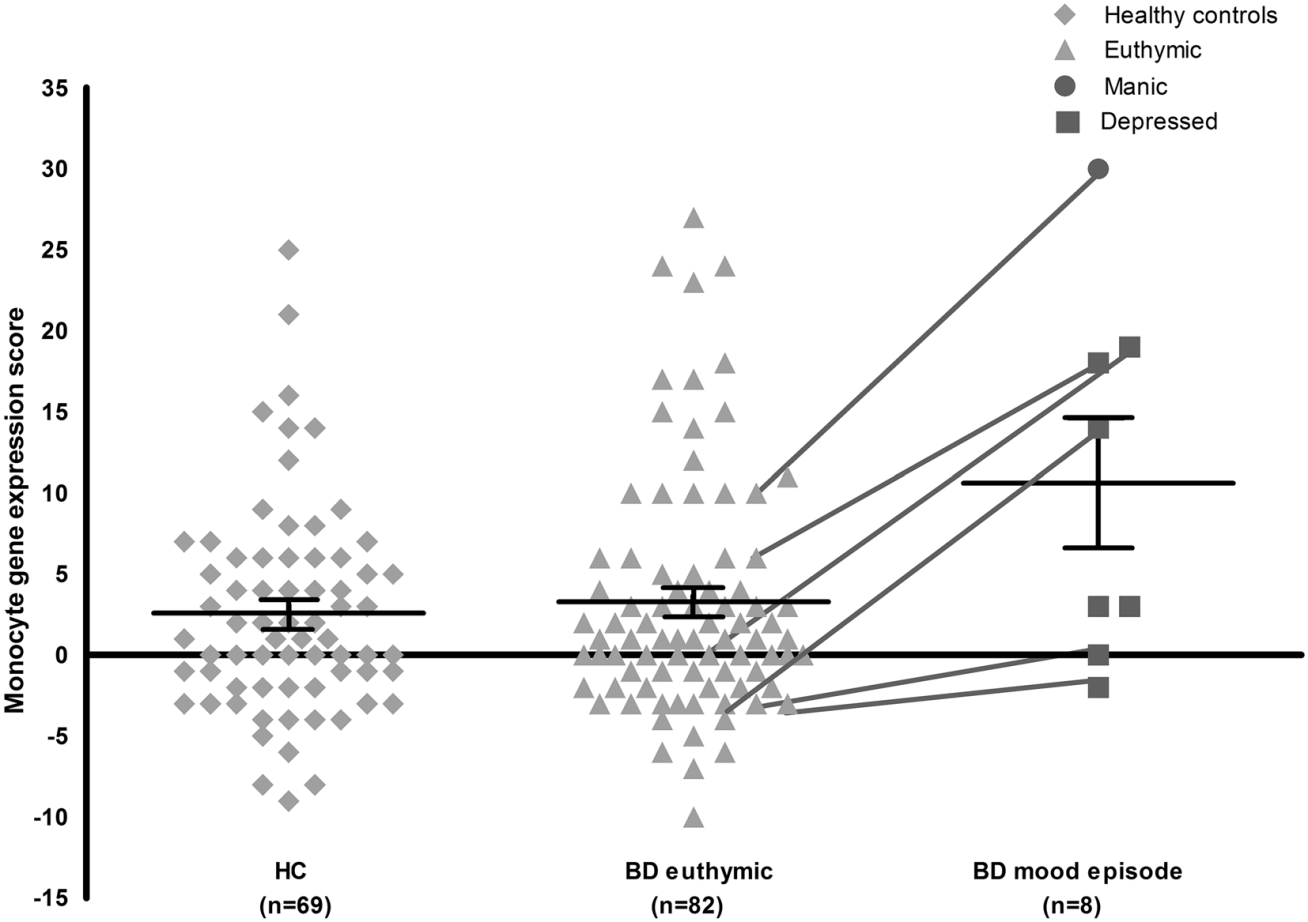
Total monocyte gene expression score of healthy controls, euthymic and mood episode BD patients (*N* = 159). *HC* healthy controls, *BD* bipolar disorder. *Black lines* represent mean and standard error of the mean per group. *Lines* connecting values from the BD-Eu and BD-Ep group represent the euthymic patients who were measured again when they had a mood episode

Table 2 shows the sample characteristics of HC, BD-Eu patients and BD-Ep patients. Figure 1 shows the total inflammatory gene expression scores in these groups. We found no significant differences between HC (2.58 ± 0.88) and BP-Eu (3.48 ± 0.84), or BD-Ep patients when they were euthymic (1.17 ± 0.94) (all *p* > .44). However, BD-Ep patients had a significantly higher total gene expression score (10.63 ± 2.58) compared to HC (*p* = .004) and BD-Eu patients (*p* = .009) and compared to their own scores when they were euthymic (*p* = .020). For the means of sub-cluster 1 score, again no significant differences were found between HC (1.13 ± 0.41), BD-Eu (1.59 ± 0.39) and BD-Ep patients when they were euthymic (0.50 ± 1.38) (all *p* > .40). BP-Ep patients (5.13 ± 1.20) again had a significantly higher sub-cluster 1 score compared to healthy controls, BD-Eu patients and compared to their own scores when they were euthymic (*p* = .002, *p* = .006 and *p* = .01, respectively). The mean sub-cluster 2 scores of HC (0.74 ± 0.28), BD-Eu patients (0.59 ± 0.26), BD-Ep patients (2.0 ± 0.81) and BD-Ep patients when they were euthymic (0.17 ± 0.94) did not differ significantly between any of the groups (all *p* > .10). When repeating the analyses adjusted for sex, age and BMI, this resulted in essentially the same results.

## Discussion

To our knowledge, the present study is the first to show an elevated inflammatory monocyte gene expression in BD patients when experiencing a mood episode, compared to both HC and euthymic BD patients. Furthermore, BD-Ep patients had an increased inflammatory gene expression than when they were euthymic. This indicates that inflammatory gene expression in BD is related to the mood state, rather than being a trait marker.

Our findings are supported by several other studies examining peripheral cytokines, where the highest levels of cytokines are found in BD patients with a mood episode, although findings in these studies were not equivocal (Barbosa et al. 2014; Brietzke et al. 2009; Cunha et al. 2008; Ortiz-Domínguez et al. 2007; Tsai et al. 2012). Serum levels of cytokines are known to follow a different pattern than monocyte gene expression (Mesman et al. 2014). Belonging to the same developmental lineage as brain microglia, monocyte activation may be more directly related to psychopathology than circulating cytokines (Beumer et al. 2012; Haarman et al. 2014b).

Previous studies from our group in different samples examining inflammatory monocyte gene expression in relation to BD found specifically the sub-cluster 2 genes to be related to a mood episode (Drexhage et al. 2010; Padmos et al. 2008) or to severity of manic symptoms (Haarman et al. 2014a). Although in our study the scores were also higher in BD-Ep patients, we did not find a significant difference in sub-cluster 2 gene score compared to HC or BD-Eu patients. This can probably be explained by the fact that we included only one manic patient, whereas in our previous studies more manic patients were included and that we used a total gene score calculation, whereas the previous studies examined the separate genes. Since sub-cluster 2 genes are associated with adhesion, cell differentiation and cell shape changes and sub-cluster 1 consists of the classic pro-inflammatory genes, it seems that in our sample having a mood episode is specifically associated with activation of the inflammatory response system.

Although our finding that an increased inflammatory gene expression is more likely to be a state than a trait phenomenon, the causality and time sequence of these associations are still difficult to interpret. Based on the present data, we cannot be sure whether an increase in inflammatory gene expression preceded the mood episode, or vice versa. In an earlier study, we showed that increased immune activation represented by peripheral markers preceded the onset of manic symptoms in MDD patients (Becking et al. 2013). The only way to examine a clear causal role for immune activation in the development of a mood episode is to measure euthymic patients multiple times prior, during and after a mood episode.

Our study has several limitations. First and most important, although the total sample consisted of 159 persons, we had only few BP-Ep patients: seven patients with a depression and only one manic patient. Since we examined only one manic patient, it is difficult to draw conclusions about inflammatory gene expression in a manic state. However, because we found already significant differences in this small group, we would encourage future studies to include more patients with a mood episode and also to assess patients repeatedly during both an episode and when euthymic. Second, all our patients were treated naturalistically, which resulted in a variety of medications known to influence inflammatory gene expression, including lithium, anticonvulsants, antipsychotics and antidepressants (Haarman et al. 2014a; Padmos et al. 2008; Rybakowski 2000; Tourjman 2012). Since these effects are typically suppressive in nature, medication may have obscured a real difference in inflammatory gene expression between BD-Eu patients and HC. However, the BP-Ep patients demonstrating significantly increased gene expression compared to both HC and BD-Eu patients used approximately the same medications, suggesting a pathophysiological cause. Third, our selection of genes was based on the study of Padmos et al. (Padmos et al. 2008) which found these specific signature genes, possibly ruling out other important genes. Finally, our study only focused on inflammatory gene expression of monocytes, which make up around 2–8 % of the total white blood cell population and is still a peripheral measurement. It would also be of interest to examine other parts of the peripheral immune system (e.g., leukocyte subsets), or more proximal factors such as microglial activation in the brain or cytokine concentrations in cerebrospinal fluid.

## Conclusions

In conclusion, our study showed that in BD patients the presence of a mood episode was associated with elevated inflammatory monocyte gene expression. This may imply that immune activation found in BD may occur in episodic patients and not in euthymic patients and can be detected in monocytes. Studies in peripheral cytokines corroborate our findings; however, our results in gene expression need to be replicated in larger samples before a firm conclusion can be drawn.

## Abbreviations

BD: bipolar disorder
BD-Ep: bipolar disorder patients with a mood episode
BD-Eu: euthymic bipolar disorder patients
BMI: body mass index
CT: comparative threshold
FC: fold change
HC: healthy control
IDS-C_30_: Inventory of Depressive Symptoms, Clinician Rated version
IDS-SR_30_: Inventory of Depressive Symptoms, Self-Rated version
MINI: Mini-International Neuropsychiatric Interview
RNA: ribonucleic acid
SD: standard deviation
YMRS: Young Mania Rating Scale
